# On the Mutational Topology of the Bacterial Genome

**DOI:** 10.1534/g3.112.005355

**Published:** 2013-03-01

**Authors:** Patricia L. Foster, Andrew J. Hanson, Heewook Lee, Ellen M. Popodi, Haixu Tang

**Affiliations:** * Department of Biology, Indiana University, Bloomington, Indiana 47405; † School of Informatics and Computing, Indiana University, Bloomington, Indiana 47405

**Keywords:** mutation rate, evolution, replication fidelity, chromosome structure, DNA polymerase errors

## Abstract

By sequencing the genomes of 34 mutation accumulation lines of a mismatch-repair defective strain of *Escherichia coli* that had undergone a total of 12,750 generations, we identified 1625 spontaneous base-pair substitutions spread across the *E. coli* genome. These mutations are not distributed at random but, instead, fall into a wave-like spatial pattern that is repeated almost exactly in mirror image in the two separately replicated halves of the bacterial chromosome. The pattern is correlated to genomic features, with mutation densities greatest in regions predicted to have high superhelicity. Superimposed upon this pattern are regional hotspots, some of which are located where replication forks may collide or be blocked. These results suggest that, as they traverse the chromosome, the two replication forks encounter parallel structural features that change the fidelity of DNA replication.

A little more than 50 years ago, Seymour Benzer published a paper entitled “On the Topology of the Genetic Fine Structure” (Benzer 1961). In that seminal report he asked “whether mutable sites are distributed at random, or whether there exist portions of the map that are unusually crowded with or devoid of sites.” The advent of accessible whole-genome sequencing has now made it possible to begin to answer Benzer’s question for the entire genome. In the study reported here, we analyzed the distribution of spontaneous base-pair substitutions (BPSs) that accumulated in a mismatch-repair defective strain of *E. coli*, MutL^–^, whose mutation rate is approximately 150 times that of its wild-type parent (Lee *et al.* 2012). To recover an unbiased spectrum of mutations we used the mutation accumulation (MA) strategy pioneered by Mukai (Mukai 1964; Halligan and Keightley 2009). The basic protocol starts with a founder individual from which parallel lines of clonal populations are established. Each line is then subjected to repeated single-individual bottlenecks. Because the effective population of each line is one, the ability of natural selection to fix or eliminate mutations is minimized. Applied to bacteria, the MA protocol consists of repeatedly streaking parallel lines for single colonies on agar plates; since colonies develop from a single cell, each passage is a one-cell bottleneck followed by approximately 28 generations of growth. Because most new mutations arise when the population is large, selection within the colony is minimal. In addition, most mutations have negligible fitness effects (Kibota and Lynch 1996; Lynch and Walsh 1998; Elena and Lenski 2003). Application of whole-genome sequencing to parallel MA lines allows an unprecedented picture of nearly unbiased mutational profiles.

Although replicative DNA polymerases make errors of the order of 10^−4^-10^−5^ per nucleotide, DNA repair pathways reduce these to 10^−9^-10^−11^ (Fijalkowska *et al.* 2012). Perhaps the most important of these pathways is the methyl-directed mismatch repair (MMR) system, which recognizes errors, destroys the newly synthesized DNA strand, and forces repolymerization templated by the old, presumably correct, DNA strand (reviewed in Marinus 2010). Eliminating MMR not only increases the error rate of replication but can also reveal the nature of the mistakes that are made by the DNA polymerases.

By sequencing the genomes of 34 parallel MA lines of the MutL^–^ strain, each of which had gone through 19 single-colony bottlenecks for a total of 12,750 generations, we identified 1625 BPSs. The mutational spectrum of these mutations has recently been described (Lee *et al.* 2012). In this report we describe and analyze the spatial distribution of these BPSs across the *E. coli* genome.

## Materials and Methods

### Bacteria strains and media

The bacterial strains and media used are described in Lee *et al.* (2012). The *E. coli* strain is PFM5, which is MG1655 *rph*^+^ Δ(*mutL*).

### MA protocol

MA lines of PFM5 were established from single colonies isolated on Miller Luria-Bertani (Miller 1992) agar plates. Subsequently, each day a well-isolated colony closest to a line drawn down the center of the plate was chosen for single-colony purification. After 19 such passages, resulting in 375 generations per line, genomic DNA purified from 34 lines was sequenced by the Beijing Genome Institute using the Illumina HiSeq2000 platform. Further details about the MA protocol, DNA extraction, sequencing, quality control, and single-nucleotide polymorphism (SNP) calling protocols are given in (Lee *et al.* 2012). Both the sequence reads and SNP calls have been deposited in the National Center for Biotechnology Information Sequence Read Archive, http://www.ncbi.nlm.nih.gov/sra, accession no. SRA054031, and the SNP calls are available at the IU Scholar Works Repository, http://hdl.handle.net/2022/15192.

### Analysis of the gap-size distribution

The distribution of the sizes of the intervals (gaps) between random events occurring either in time or in space is described by the exponential distribution; the mean of this distribution is the total length of time or distance divided by the number of events (Rice 1995). Thus, the mean gap-size predicted for the BPS data is the length of the chromosome, 4640 kb, divided by the number of BPS, 1625, and equals 2.86 kb. However, analysis of the distribution of the gap-sizes was complicated by the fact that the location of BPSs in repeated sequences [insertion sequence (IS) elements, rRNA operons, and other smaller repeat sequences] could not be defined and thus were excluded from the data. To account for this complication, we eliminated all the gaps that included IS or rRNA operons (other repeated sequences are too small to bias the results). This adjustment, which left 1581 BPSs over 4284 kb for a mean gap-size of 2.71 kb, made little difference—with or without the repeat elements the distribution was significantly different from the expected distribution. Removal of these repeat elements did not change the pattern of mutational density across the chromosome (see Supporting Information, Figure S1A).

### Bin analysis

The 1625 BPSs that accumulated in the MutL^−^ strain were collected into 46 bins, each bin approximately 100 kb in size, starting at the origin of replication (see Table S1). The mean number of BPSs per bin was 35.3 with a variance of 95.2. The number of bins was chosen to be 46 because that number: (1) is close to the square-root of the sample size, a common “rule of thumb” for choosing the number of intervals for a histogram; (2) describes the data clearly (see Figure 5); and, (3) divides into the total number of nucleotides in the genome with an acceptably small remainder. However the mutation-density pattern was stable against changes in bin-size from about 50 kb (91 bins), which gave a mean number of BPSs per bin of 17.9 with a variance of 35.7, to about 220 kb (21 bins), which gave a mean number of BPS per bin of 77.4 with a variance of 307 (see Figure 5). The pattern was also unaffected by a 50% displacement of the bin starting point and was preserved when the binning started at the traditional zero point of the *E. coli* chromosome instead of at the origin of replication (see Figure S2).

### Wavelet transformation

Fourth-order Daubechies wavelet transforms were performed on the binned mutational data using the built-in program in Wolfram Mathematica 8.

### Correlations and linear regressions with chromosomal features

Data describing various chromosomal features were obtained from the referenced sources (see Table 1 and Table S2) and collected into the same 46 bins used for the mutational data. Discrete data (such as number of transcription factor binding sites) were summed. Qualitative data (such as gene expression) were both summed and averaged and correlations with the mutational data calculated for each result; in every case the average value in each bin gave the best correlation. Pearson’s product-moment correlation coefficient ρ_p_, was calculated for each of the 38 features listed in Table 1 and Table S2 compared with the binned mutation values. The product-moment correlation tests for a linear relationship; to ensure that no correlations were missed, we also calculated Spearman’s rank correlation coefficient (corrected for ties), which is distribution free. With one exception, the rank correlation coefficients were similar to or less than the linear ones, indicating the relationships were better described by linear comparisons. The single exception was the relationship between the mutations and the number of relaxation-induced genes, which had a non-significant (*p* and *q* = 0.09) rank correlation coefficient of 0.235 but a linear coefficient of 0.077 (see Table S2).

The significance of the correlation coefficients was evaluated from the two-tailed t-distribution [using the web-based tools at http://www.wessa.net/ (Wessa 2012)]. The resulting *p*-values give the probable false positive rate, *i.e.*, the probability that a correlation that is actually zero will have a coefficient equal to or greater than the given value; the traditional *p*-value cutoff for accepting the correlation is 0.05. We also computed *q*-values, which are a measure of the false discovery rate commonly used when multiple comparisons are being simultaneous considered (Storey and Tibshirani 2003). These were computed using the Qvalue program (Storey and Tibshirani 2003) with the false discovery rate set to 0.05. The *q*-values give the probability that a correlation considered to be significant is actually zero; applied to our results, among the eleven correlations with a *q*-value ≤ 0.05 (see Table 1) the probable number of false-positive results is less than one. The *q*-values so calculated are conservative because many of the comparisons were not independent (*e.g.*, up-regulated and down-regulated genes are subsets of the total number of affected genes) and so the number of comparisons is inflated.

Linear regressions were performed using the built-in function of the XLSTAT package (Addinsoft SARL) for Microsoft Excel (Microsoft Corp). The best model was chosen by minimizing Akaike’s Information Criterion (Akaike 1973).

## Results

**Figure 1:**
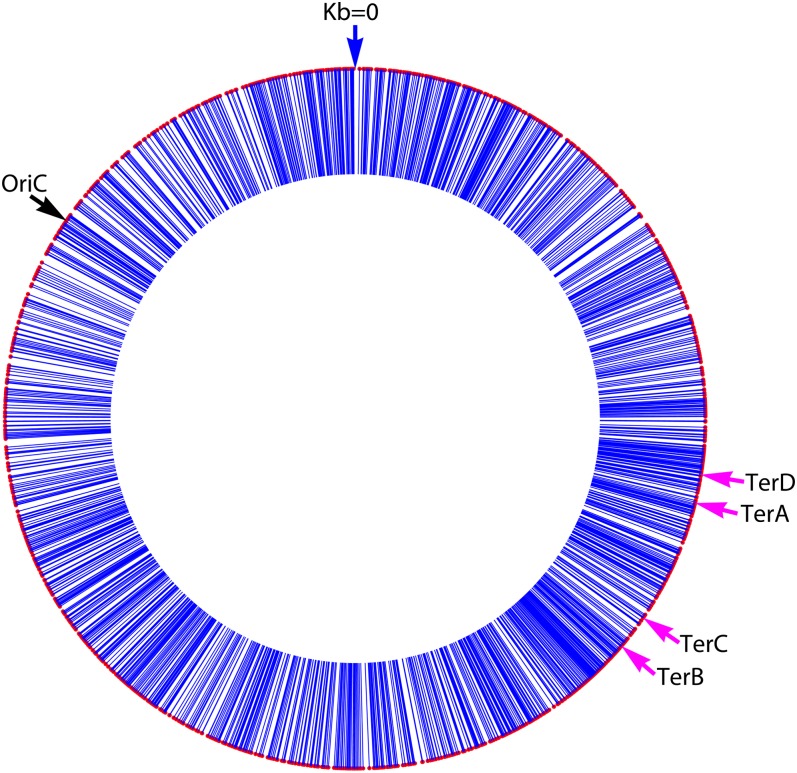
The distribution of BPSs across the genome. The 4640-kb *E. coli* chromosome is shown with the traditional zero point at the top. Each blue line with a red cap indicates the position of a BPS that accumulated in the MutL^−^ strain; the thickness of each line is equivalent to approximately one kb, centered on the position of the BPS. OriC, the origin of replication at 3924 kb; TerD, TerA, TerC, and TerB, strong termination sites at 1279 kb, 1340 kb, 1607 kb, and 1682 kb, respectively (Duggin and Bell 2009). Clockwise (rightward) moving forks are halted at TerC or TerB and counterclockwise (leftward) moving forks are halted at TerA or TerD.

**Figure 2:**
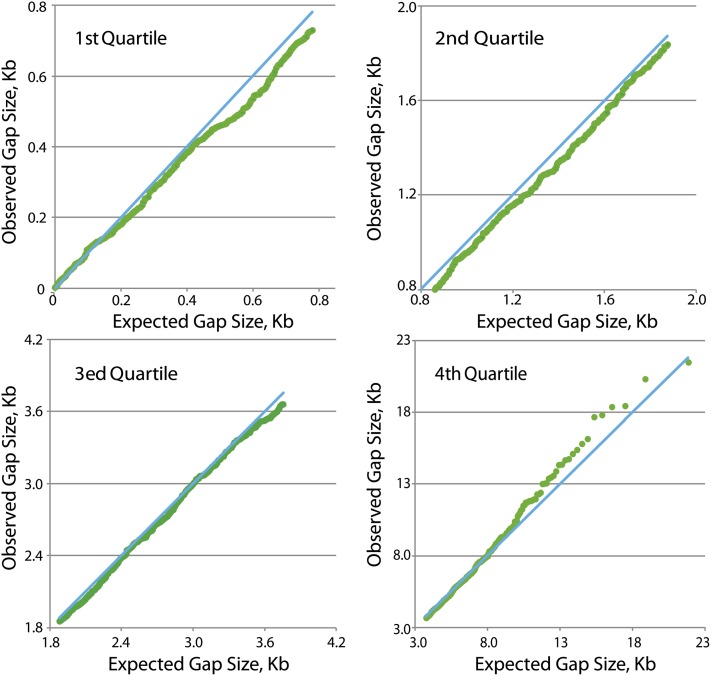
The distribution of the gaps between BPSs. Shown are the four quartiles of a quantile-quantile (Q-Q) plot (Rice 1995) of the observed sizes of the intervals (gaps) between BPSs *vs.* the sizes predicted by an exponential distribution. For this analysis, gaps that contained large repeat elements have been removed (see *Materials and Methods*); this procedure left 1581 BPSs distributed over 4284 kb of the chromosome, giving a mean gap size of 2.71 kb. The observed distribution is significantly different than expected (χ^2^ ≈10^4^; *p* ≪ 0.0001).

The *E. coli* strain used for this study has a nonpolar deletion of the *mutL* gene, which encodes MutL, a protein required for MMR (Lee *et al.* 2012). A total of 1625 BPSs were recovered from the MutL^−^ strain and their spatial distribution across the 4640 Kb *E. coli* genome is shown in Figure 1. Although the distribution appears to be random by eye, it is not. If the BPSs were distributed at random, the size of the gaps between adjacent BPSs would have an exponential distribution. However, the actual distribution is significantly different from expected (χ^2^ ≈10^4^; *p* ≪ 0.0001). Below a gap size of about 0.2 kb the observed distribution fits the expected distribution fairly well, but gap-sizes began to deviate from expected at values greater than 0.2 kb (Figure 2). Over most of the range the gap sizes are smaller than expected, meaning that the mutations are clustered.

**Figure 3:**
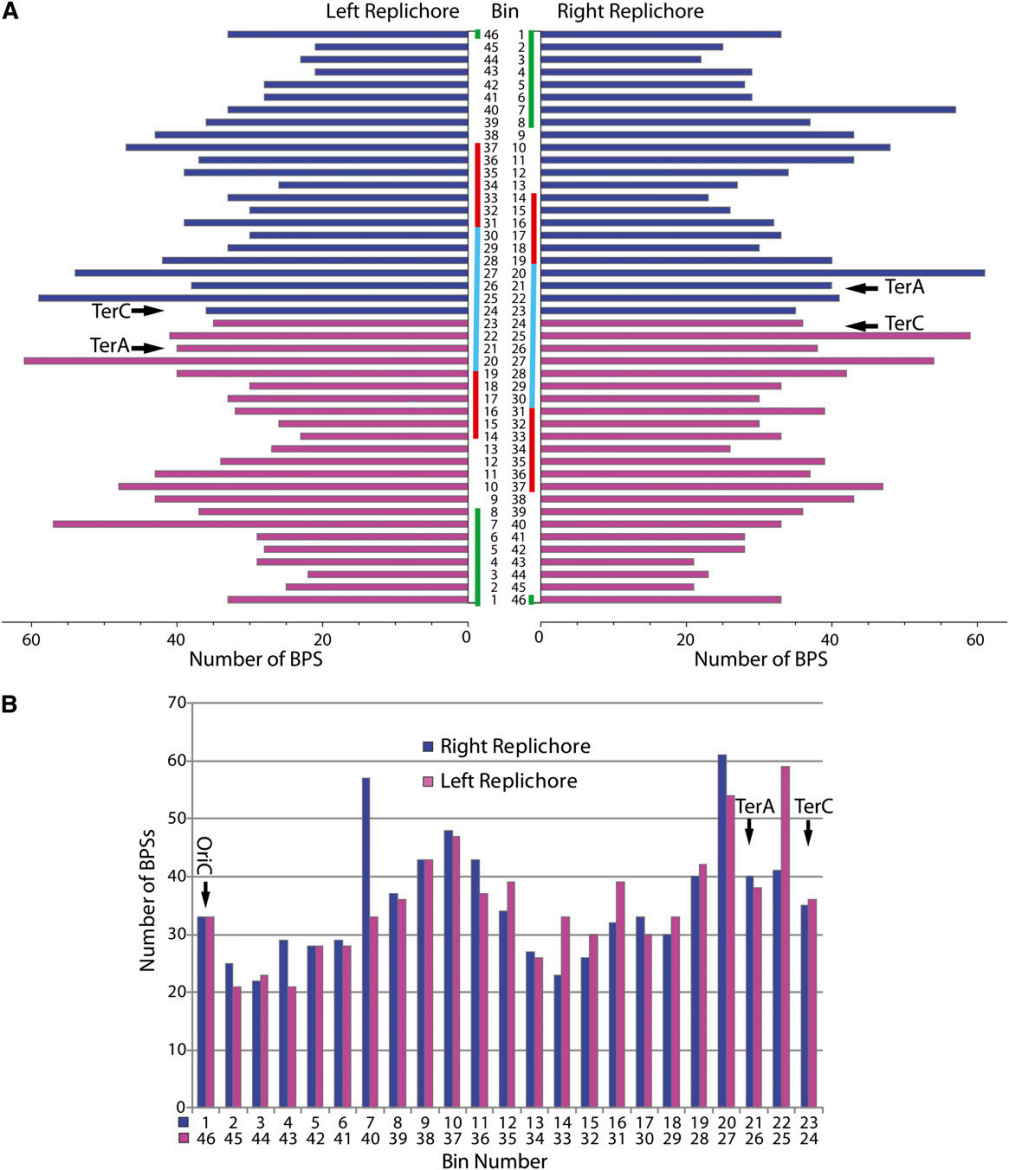
The distribution of binned BPSs across the genome. (A) The 1625 BPSs that accumulated in the MutL^−^ strain collected into 46 bins, each bin approximately 100 kb in size, starting at the origin of replication. Each side of the histogram displays the binning in opposite directions, reproducing the movement of the two replication forks as if each continued across the whole chromosome (*i.e.*, the lower left quadrant is the inverted mirror image of the upper right quadrant, and *vice versa*); the color changes from blue to magenta at the midpoint of the chromosome. The strong termination sites, TerA and TerC, in bins 21 and 24, respectively, are indicated; not indicated are the alternative strong termination sites, TerD in bin 20 and TerB in bin 24. The four MDs defined by the efficiency of recombinational exchange within each domain (Niki *et al.* 2000; Valens *et al.* 2004) are indicated: green, Ori MD; cherry, left MD and right MD; cyan, terminal MD. (B) The bins in (A) reoriented so to directly compare the mutational pattern of the two replichores. Note that the Ter sites are not symmetrically oriented with respect to the origin; the midpoint of the chromosome lies between bins 23 and 24, close to TerC. Thus, the peaks in mutational density in bins 20 and 25 surround the terminal region bounded by TerA and TerC, whereas the peak in bin 27 is well outside of this region.

A more interesting departure from random is revealed by the locations of the mutations. In Figure 3A the 1625 BPSs are collected in 46 bins, each bin approximately 100 kb wide, starting at the origin of replication. The left and right sides of Figure 3A display the same data collected in opposite directions, reproducing the movement of the two replication forks as they traverse the chromosome, but arranged as if each fork continued around the chromosome and back to the origin (*i.e.*, the lower left quadrant is the inverted mirror image of the upper right quadrant, and *vice versa*); the color changes from blue to magenta at the midpoint of the chromosome. Figure 3A reveals that the mutations are distributed across the genome in a large-scale, periodic pattern that is repeated nearly in mirror-image in the two replichores. The distributions of the mutations per bin between the two replichores are highly correlated (Pearson’s correlation coefficient, ρ_p_, = 0.701, *p* = 0.0002) (Figure 3B); thus, the pattern appears to reflect genomic features that affect the fidelity of the two replisomes in parallel as they move from the origin to the terminus. Starting from OriC (the top of Figure 3A) the density of mutations in each replichore drops for about 500 kb (bins 2−6, 45−42), then increases, reaching a peak at about 1000 kb from OriC (bins 10 and 37). The density then drops to another low at about 1400 kb from OriC (bins 13−15, 34−32) before climbing to a second peak at about 2100 kb from OriC (bins 20, 25), which is immediately before the terminus region. The density of mutations drops again at the point where each fork would pass the strong termination sites TerD and TerA (located in bins 20 and 21, respectively) for the clockwise-moving fork and TerB and TerC (both located in bin 24) for the counter-clockwise moving fork. Within the inner termination region (bins 21−24) the mutation density is relatively constant and close to the average across the whole genome.

The pattern shown in Figure 3A is unlikely to be obtained by chance. A random distribution of 1625 mutations in 46 bins is expected to be Poisson with a variance equal to the mean of 35.3. But the actual distribution has a variance of 95.2 and differs significantly from the Poisson (χ^2^ = 143, *p* ≈ 10^−12^). We performed 1000 Monte Carlo simulations of 1625 mutations reproducing the MutL^–^ mutational spectrum, distributed at random across the genome and then gathered into 46 bins. The variances of the 1000 distributions averaged 35.7 and ranged from 15.3 to 63.6. Thus, the maximum variance of 1000 random distributions was only two-thirds that of the actual data. Comparisons of a subset of 100 of the simulations to the actual data showed that all the distributions were significantly different from the symmetrical pattern shown in Figure 3A (the minimum χ^2^ = 103, *p* = 2 × 10^−6^).

**Figure 4:**
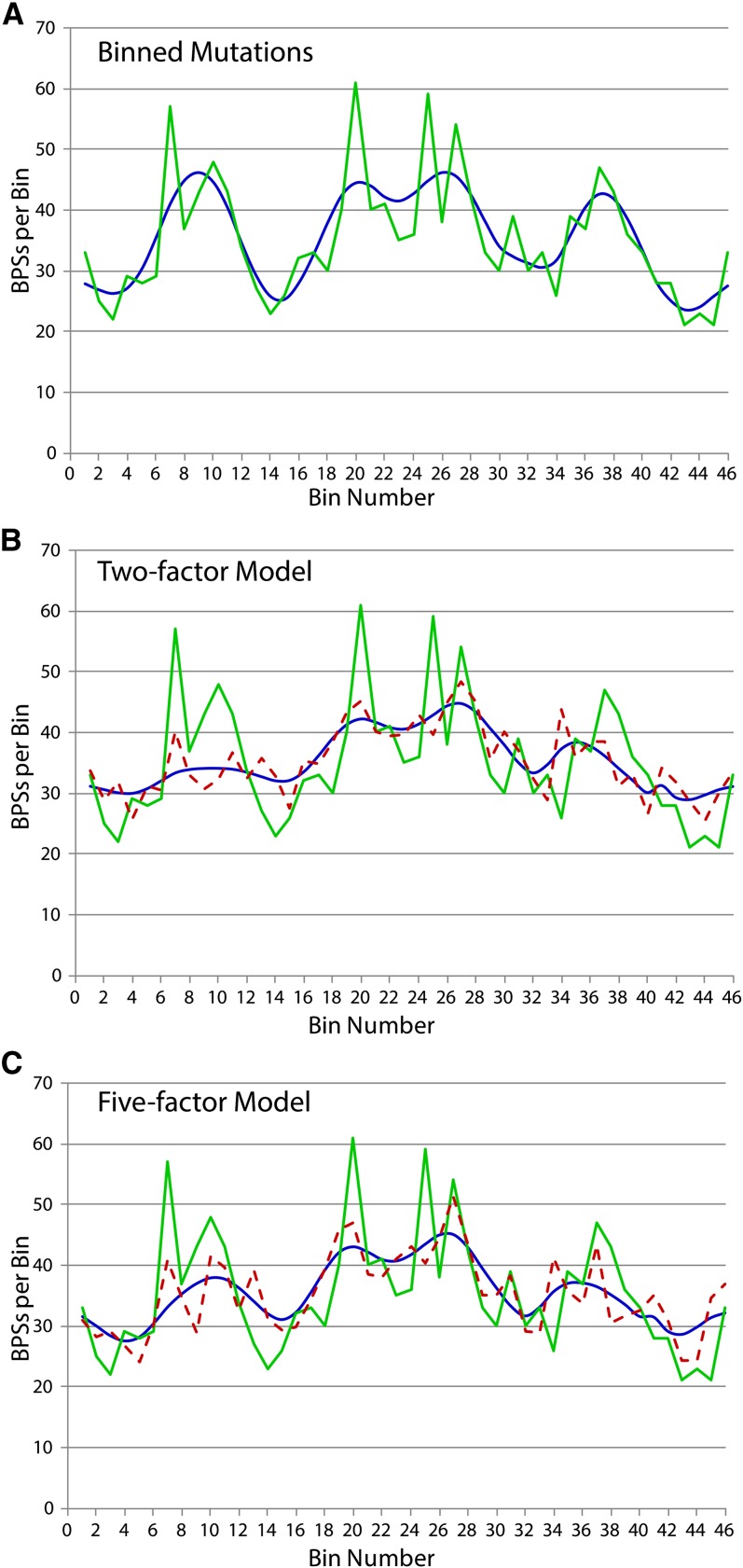
Wavelet transformations of the mutational distribution. (A) The numbers of BPSs in each of 46 bins are plotted in green with the bins arranged proceeding clockwise across the chromosome starting and ending at the origin of replication (OriC). The Daubechies wavelet transform is plotted in blue. (B) Two-factor model. A linear regression of the number of mutations against 10 chromosomal features significantly correlated with the mutational data (see Table S3) produced an optimal two-factor model. The model is: mutations = 16 + (7.8 × HU) + (1.5 × FIS), where HU indicates the HU response per gene minus *hupAB* and FIS indicates the number of genes up-regulated in a Fis^−^ mutant (see Table 1). For this model, r^2^ = 0.335, *p* = 0.0002. The numbers of mutations per bin predicted by this model (dashed, magenta) and the corresponding Daubechies wavelet transform curve (blue) are compared to the observed numbers of mutations per bin (green). (C) Five-factor model. To produce the five factor model, a linear regression of the number of mutations was performed against the ten features that were used to generate the two-factor model plus seven additional features that had positive or negative correlation coefficients with the mutational data of ≈ 0.2 (see Table S3). The model is: mutations = 68 – (97 × CAI) + (5.0 × HU) + (1.0 × FIS) + (0.9 × RR) – (0.8 × H-NS), where HU and FIS are defined as previously; CAI indicates the average gene CAI; H-NS indicates the number of genes down-regulated in an H-NS mutant; and RR indicates the number of relaxation repressed genes. For this model, r^2^ = 0.435, *p* = 0.0003. The numbers of mutations per bin predicted by this model (dashed, magenta) and the corresponding Daubechies wavelet transform curve (blue) are compared to the observed numbers of mutations per bin (green).

**Figure 5:**
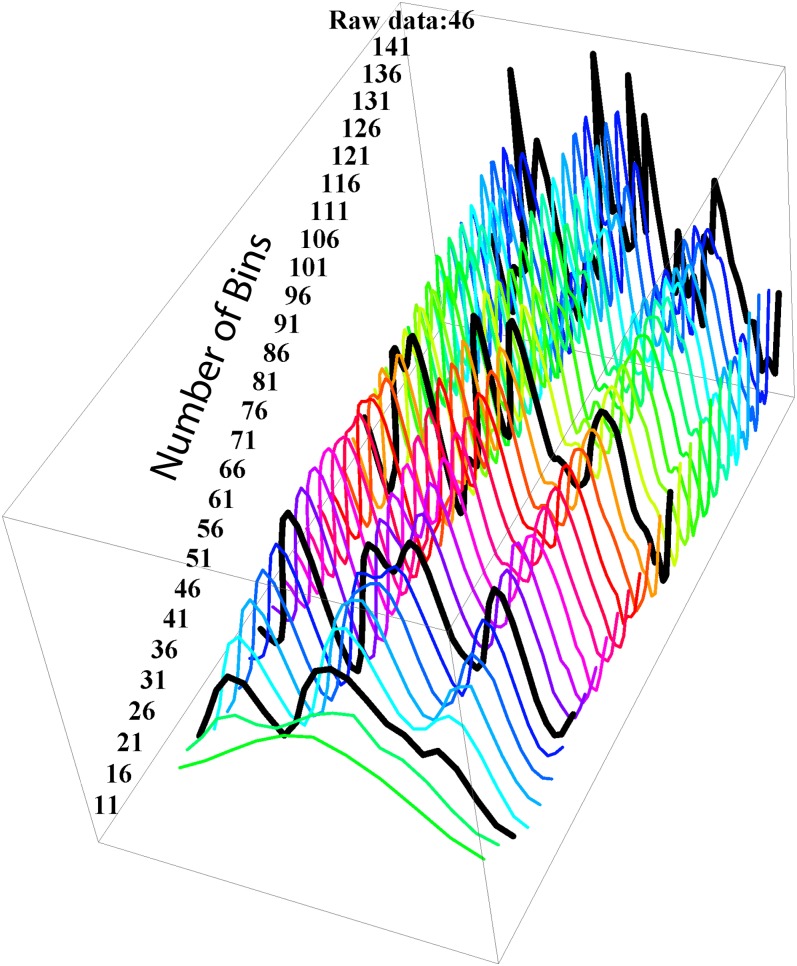
The stability of wavelet transform against changes in bin size. Daubechies wavelet transforms were applied to the binned data with the number of bins per chromosome varying from 11 to 141 (corresponding to bin sizes of 422 kb to 33 kb), resulting in the curves plotted here. Black lines indicate 21, 46, and 91 bins, with the terminal black line indicating the raw, untransformed 46-bin data.

The underlying symmetrical pattern of the mutational density is revealed clearly by the Daubechies wavelet transform shown in Figure 4A. Applying this wavelet smoothing across continuously varying bin sizes preserved the underlying histogram structure in the range of bin sizes from roughly 50 kb (91 bins) to 220 kb (21 bins), with degradation outside that range (Figure 5). The Daubechies wavelet transform also reveals two regional hotspots, one 600−700 kb from the origin (bin 7) in the right replichore and one before the terminal region in the left replichore (bin 27), that do not fit the symmetrical pattern.

We have attempted to correlate the periodic pattern shown in Figure 3A with various features of the chromosome gathered into the same 46 bins. Table 1 gives the significant correlations; nonsignificant correlations are listed in Table S2. The MutL^−^ mutational spectrum is dominated by A:T > G:C transitions (Lee *et al.* 2012); thus, the number of mutations and the A:T content of each bin are correlated (Table 1). However, the mutational pattern is not simply due to the distribution of A:T base pairs across the genome; if the numbers of mutations at A:T and G:C base pairs are normalized by the A:T and G:C content of each bin, the pattern does not differ significantly from the one shown in Figure 3A (χ^2^ = 0.3, *p* = 1.00; see Figure S1B).

**Table 1:** Correlations of the numbers of mutations per bin with various genomic features

	Pearson’s Product-Moment Correlation Coefficient*^a^*			
Feature	ρ_p_	*p*	*q*	Data Reference
Sequence features				
A:T content	0.325	0.027	0.045	NCBI*^b^*
Gene expression				
Average gene CAI	−0.373	0.011	0.023	(Puigbo *et al.* 2008)
Genomic structural features				
No. of HU sensitive genes	0.141	0.347	0.217	(Berger *et al.* 2010)
No. of genes up-regulated in HU^−^ mutant	−0.266	0.074	0.096	(Berger *et al.* 2010)
No. of genes down-regulated in HU^−^mutant	0.387	0.008	0.022	(Berger *et al.* 2010)
HU response per gene*^c^*	0.397	0.006	0.022	(Berger *et al.* 2010)
HU response per gene minus *hupAB*	0.455	0.001	0.006	(Berger *et al.* 2010)
Gyrase binding distribution*^d^*	−0.360	0.007	0.022	(Jeong *et al.* 2004)
No. of gyrase sensitive genes*^d^*	0.382	0.009	0.022	(Jeong *et al.* 2004)
No. of genes up-regulated in *gyrA* mutant	−0.110	0.466	0.282	(Jeong *et al.* 2004)
No. of genes down-regulated in *gyrA* mutant	0.481	0.001	0.006	(Jeong *et al.* 2004)
No. of FIS sensitive genes (mid-log)*^e^*	0.365	0.013	0.024	(Blot *et al.* 2006)
No. of genes up-regulated in Fis^−^ mutant	0.457	0.001	0.006	(Blot *et al.* 2006)
No. of genes down-regulated in Fis^−^ mutant	0.059	0.698	0.358	(Blot *et al.* 2006)
SeqA binding sites*^f^*	−0.322	0.029	0.045	(Sanchez-Romero *et al.* 2010)

NCBI, National Center for Biotechnology Information; CAI, codon adaptation index.

a ρ_p_ is the correlation coefficient; *p* is a measure of the false positive rate; *q* is a measure of the false discovery rate appropriate for evaluating multiple comparisons (see *Material and Methods*).

b The reference genome sequence was NC_000913.2 (MG1655).

c HU response per gene is the average ratio of gene expression in a wild-type strain to that in a *hupAB* mutant strain based on microarray analysis. *hupA* and *hupB* encode the two subunits of HU and are strongly downregulated in the *hupAB* mutant strain.

d Data obtained from http://www.ncbi.nlm.nih.gov/geo/. Gyrase binding distribution is based on ChIP-chip microarray experiments and has been normalized to the number of genes analyzed. Gyrase sensitive genes had a ratio of gene-expression in a wild-type strain to that in a *gyrA* mutant either ≥ 1.5 (up-regulated) or ≤ 0.975 (down-regulated). The *gyrA* mutant has reduced gyrase activity (Jeong *et al.* 2004).

e FIS-sensitivity is the ratio of gene expression in a wild-type strain to that in a Δ*fis* mutant strain, based on microarray analysis of cells in mid-exponential phase.

f SeqA binds to hemi-methylated GATC sequences; one of its functions is to regulate replication initiation. The data are from ChIP-chip analyses.

Gene expression, as measured by the number of genes that are transcriptionally active under a variety of conditions, has been found to oscillate across the genome with periodicities of 600−800 kb (Allen *et al.* 2003, 2006). These oscillations, particularly in the terminus region, correspond roughly to some of the peaks and valleys in the mutational density pattern. However, although the distribution of mutations is symmetrical in the two replichores, the distribution of gene expression is not, and the overall correlation between the number of mutations and gene expression values used in Allen *et al.* (2006) is low (Table S2). Gene expression measured in a number of other studies was also poorly correlated with the pattern of mutational density reported here (Table S2). There is a significant negative correlation between the number of mutations and the average gene Codon Adaptation Index (CAI) (Table 1); because CAI is indicative of gene expression, this correlation suggests that mutation rates are low in regions of the chromosome that contain a high density of highly expressed genes. However, the average CAI and the A:T content per bin are negatively correlated (ρ_p_ = −0.744, *p* ≪ 0.0001), so it is not clear which of these two is the most important causal factor.

In a recent study, Martincorena *et al.* (2012) analyzed the numbers of synonymous BPSs in 2659 genes in 34 diverged *E. coli* genomes. The distribution of synonymous diversity values (related to BPSs per gene) across the genome has some similarity to the mutational pattern we observe, again particularly in the terminus region (see Figure 1A in Martincorena *et al.* 2012). The synonymous diversity values in the right replichore declined near the positions of TerD and TerA, the same point at which the mutational densities reported here also declined. In the left replichore, the synonymous diversity values declined at about 2.1 Mb, well before the replisome would reach TerB and TerC but still within the general terminus region. There may be other correlations between the distribution of synonymous BPSs and the mutational patterns we observed that are yet to be discovered by further analysis of the data. However, the examples given by Martincorena *et al.* (2012) of a cold spot at 848 kb and a hot spot extending from 4314 kb to 4334 kb are not corroborated by our data; when the number of BPSs that we observed in each of these regions was normalized to size of the region in nucleotides, the density of BPSs was lower in the “hot” spot than in the “cold” spot. In addition, the mutation-density distribution reported here is not correlated with the gene expression data reported in Martincorena *et al.* (2012) (see Table S2), whereas they reported a negative correlation of their synonymous diversity values with gene expression (Martincorena *et al.* 2012).

The *E. coli* chromosome has been divided into four macrodomains (MDs) defined by the efficiency of recombinational exchange within each domain (Niki *et al.* 2000; Valens *et al.* 2004); the approximate borders of the MDs are shown on the histograms in Figure 3A and Figure S2. The mutational density pattern does not correspond well to the MDs because, unlike the mutation pattern, the MDs are not symmetrically located in the two replichores. In general, mutational densities reported here tended to be low in the Ori MD and in the right and left MDs, and greater in the terminal MD and in the nonstructured regions that flank the Ori MD (Figure 3A and Figure S2). Comparative studies of diverged strains have also indicated that mutation rates are greater at the terminus than at the origin (Touchon *et al.* 2009; Martincorena *et al.* 2012); although this conclusion is not well supported by experimental data in bacteria (Ochman 2003), it is supported by studies of *Saccharomyces cerevisiae* (Agier and Fischer 2012).

The *E. coli* chromosome is organized into supercoiling domains that are constrained by nucleoid-associated proteins (NAPs) (Travers and Muskhelishvili 2005). Long-range periodicities in supercoiling-sensitive gene expression have been found across the *E. coli* genome and correlated to gyrase binding sites (Jeong *et al.* 2004). Clusters of supercoiling-sensitive genes have also been found at the edges of the Ter domain, corresponding to binding sites for two NAPs, FIS and H-NS (Ussery *et al.* 2001; Scolari *et al.* 2011). The histone-like protein, HU, does not have well defined binding sites, but genes that respond positively to loss of HU are located toward OriC and genes that respond negatively to loss of HU are located toward the terminus (Sobetzko *et al.* 2012). The mutational patterns reported here correspond in part to these patterns (Table 1). The distribution of BPS-density is positively correlated to the distributions of genes that are up-regulated in the absence of FIS (presumably genes that are repressed by FIS binding), genes that are down-regulated in a gyrase mutant (presumably genes that are activated by negative supercoiling), and genes that are down-regulated in the absence of HU (presumably genes that are activated by HU binding). The last two correlations may reflect the fact that HU and gyrase act together to promote superhelicity (Malik *et al.* 1996) and suggest that the mutation rate is increased in regions of the genome with high superhelical density.

To find a predictive model for the observed pattern of mutational densities, we performed linear regressions with 10 chromosomal features that were significantly correlated with the binned mutational data (positive or negative correlation coefficients > 0.3; see Table S3). Just two features, the average HU response (HU-activated genes) and the number of FIS-repressed genes, account for 33.5% of the variation in the mutational data (Figure 4B). The inclusion of seven additional features that are less well correlated with the mutational data (positive or negative correlation coefficients ≈ 0.2; see Table S3), increased the predictive power of the model. The five-factor model (Figure 4C), which accounts for 43.5% of the variation in the mutational data, adds as a positive factor the number of relaxation repressed genes and as negative factors the average gene CAI and the number of genes down-regulated in a H-NS mutant. Adding more features contributed little to the predictive value of the resulting models.

Although both the two- and five-factor models predict the general shape of the mutational pattern, both also fail to account for the high density of mutations in certain bins, particularly 7, 20, and 25. If the values in these bins are replaced by the average, linear regressions will yield models accounting for up to 50% of the variation in the mutational data. It is, of course, possible to fit the mutational pattern with nonlinear combinations of many parameters (*e.g.*, a 35-parameter nonlinear equation achieves an 80% predictive value), but the biological meaning of such complex combinations of parameters is not obvious.

## Discussion

The BPSs that accumulated in the MutL^−^ strain fell into wave-like patterns of increasing and decreasing mutational densities that are symmetrically arranged in the two replichores (Figures 3 and 4). This pattern suggests that as the chromosome is replicated, the DNA polymerases encounter parallel chromosomal features that change the fidelity of replication. In general the mutational density was high in regions of the chromosome where gene expression is responsive to NAPs. Specifically, the mutation density was positively correlated to the density of HU-activated genes and FIS-repressed genes (Table 1, Figure 4B). Both of these NAPs are strong determinants of chromosome architecture; HU contributes to negative superhelicity by constraining supercoils and compacting the DNA into nucleosome-like particles, whereas FIS also constrains supercoils but produces branches in the supercoiled DNA (Schneider *et al.* 2001). We interpret the correlation of the mutational density to regions where genes respond to these NAPs to be due to the structure of the chromosome, not to the gene expression *per se*. That is, the gene response is an indicator of regions of high chromosomal structure, and these regions result in high mutation rates when encountered by the replicating polymerases.

Additional evidence for this interpretation comes from the correlation of the mutational density with genes that are down-regulated in a *gyrA* mutant (presumably genes that are activated by negative supercoiling) (Table 1) and the inclusion of relaxation-repressed genes as a positive factor in the 5-factor model (Figure 4C). This hypothesis was not supported by the inclusion of the number of genes down-regulated in an H-NS mutant (presumably H-NS-activated genes) as a negative factor in the 5-factor model. However, H-NS, which also compacts DNA, acts antagonistically to FIS in gene regulation (Travers and Muskhelishvili 2005), suggesting that FIS and H-NS may have different effects on chromosome structure. All of these NAPs have patterned distributions and contribute to the spatial and temporal regulation of the superhelicity of the chromosome (Sobetzko *et al.* 2012). Recently, the density of the somatic mutations in human cancer cells was found to be correlated with chromatin organization (Schuster-Bockler & Lehner 2012).

On the basis of previous studies (Hudson *et al.* 2003; Martincorena *et al.* 2012), we expected mutational density to be correlated either negatively or positively with gene expression. However, the results were conflicting and no convincing correlations with gene expression were found (Table S2). Mutational density was significantly negatively correlated with the average gene CAI, and this feature appeared in the 5-factor model (Figure 4C). Because high CAI is indicative of high gene expression, this correlation suggests that mutation rates are low in regions of highly expressed genes. Our failure to confirm this with gene-expression data may mean that the data that we analyzed was inappropriate to the conditions of our experiments.

To reveal errors made during replication, we used a strain defective in MMR. MMR is initiated when a homodimer of MutS recognizes and binds a mismatch. MutS then recruits MutL and together they activate the downstream events that repair the mismatch (Marinus 2010). For the experiments reported here we inactivated MMR with a nonpolar knockout mutation of MutL. We chose to eliminate MutL instead of MutS because we thought it possible that loss of MutS has additional unwanted phenotypes. Examples are: MutS may travel with the replication fork via its interaction with the beta-clamp (Lopez de Saro *et al.* 2006; Pluciennik *et al.* 2009); MutS alone can block recombination between diverged DNA *in vitro* (Worth *et al.* 1994); and certain *mutS* mutant strains have been reported to have an unexplained loss of viability (Boe 1990). Previous studies have found little difference in the mutation rates or mutational spectra of *mutL* and *mutS* mutant strains (Schaaper and Dunn 1987). However, loss of MutL may also have unwanted phenotypes. After binding the mismatch, MutS leaves the site and rapidly slides along the DNA; another MutS homodimer may then repeat the process (Cho *et al.* 2012). In the absence of MutL and with the resulting persistence of the mismatch, this process might continue for some time and possibly affect the topology of the chromosome and/or interfere with replication fork progression. Although this process might cause mutations to be clustered, it is difficult to see how it could account for the symmetrical pattern of mutational density that we observed unless the initiating mismatches themselves had a symmetrical spatial pattern.

The data reported here suggest that as they traverse the chromosome, the two replication forks encounter in parallel regions of high DNA structure that result in a decrease in the fidelity of replication. Interestingly, sharp peaks of mutation density are located nearly symmetrically around the terminus region, close to the strong termination sites TerD and TerA on the right replichore and TerB and C on the left replichore (Figures 3A and 4A). Ter sites create a replication fork trap that prevents the two forks from leaving the terminus region; the clockwise moving fork is halted at TerC or TerB and the counterclockwise moving fork is halted at TerA or TerD [additional Ter sites are positioned as backups (Duggin and Bell 2009)]. The *E. coli* terminus region is unique both structurally [with high curvature (Pedersen *et al.* 2000) and low superhelicity (Sobetzko *et al.* 2012)] and genetically [with few genes and a high recombination rate (Louarn *et al.* 1994)]. The arrangement of the peaks in mutational density suggests that a spike in the mutation rate may occur after a replisome traverses the terminus and is blocked at the relevant Ter site, or when the two replisomes collide at a Ter site.

Blocking or impeding the replication fork by collisions or by DNA structural features could impact replication fidelity in several ways. For example, if the replicating polymerase stalls and disengages from the DNA, one of the cell’s error-prone DNA polymerases could gain access and synthesize a tract of error-containing DNA. Alternatively, DNA repair pathways other than MMR may be less active in regions of highly structured DNA than in less structured regions, or highly structured DNA may be more susceptible to spontaneous damage. Further research is needed to resolve these questions. The simple linear models predict that, in general, mutation rates increase when the replication fork encounters regions of high structure and, possibly, low gene expression. Superimposed upon this pattern are regional hotspots, some of which appear to be correlated with replication fork blocks or collisions, and some of which remain a mystery.

## Supplementary Material

Supporting Information
