# Supplementary material for: On the Mutational Topology of the Bacterial Genome
Source: G3 (Bethesda). 2013 Mar 1;3(3):399–407. doi: 10.1534/g3.112.005355 (PMC3583449; doi:10.1534/g3.112.005355)
Supplement: Supporting Information [file supp_3_3_399__index.html]

Supporting Information 

# On the Mutational Topology of the Bacterial Genome

## Supporting Information for Foster *et al.*, 2013

**Files in this Data Supplement:**

- Supporting Information - Figures S1 and S2 and Tables S1-S3 (PDF, 264 KB)
- Figure S1 - The stability of the pattern of BPS density to normalization (PDF, 157 KB)
- Figure S2 - The stability of the pattern of BPS density after bin displacement (PDF, 115 KB)
- Table S1 - The nucleotide coordinates of forty-six equally-sized bins starting at the origin of replication (PDF, 74 KB)
- Table S2 - Non-significant correlations of the numbers of mutations per bin with various genomic features (PDF, 117 KB)
- Table S3 - Genomic features used in linear regressions with the mutational data (PDF, 89 KB)
