# Supplementary material for: On the Mutational Topology of the Bacterial Genome
Source: G3 (Bethesda). 2013 Mar 1;3(3):399–407. doi: 10.1534/g3.112.005355 (PMC3583449; doi:10.1534/g3.112.005355)
Supplement: Supporting Information [file supp_3.3.399_005355SI.pdf]

G3 · Genes | Genomes | Genetics

On the Mutational Topology of the Bacterial Genome

Patricia L. Foster*, Andrew J. Hanson[§], Heewook Lee[§], Ellen M. Popodi*, Haixu Tang[§]

*Department of Biology, and [§]School of Informatics and Computing, Indiana University, Bloomington, IN, USA, 47405

Corresponding Author:
Patricia L. Foster
Department of Biology
1001 East Third Street
Indiana University
Bloomington, IN 47405
812-855-4084
plfoster@indiana.edu

DOI: 10.1534/g3.112.005355

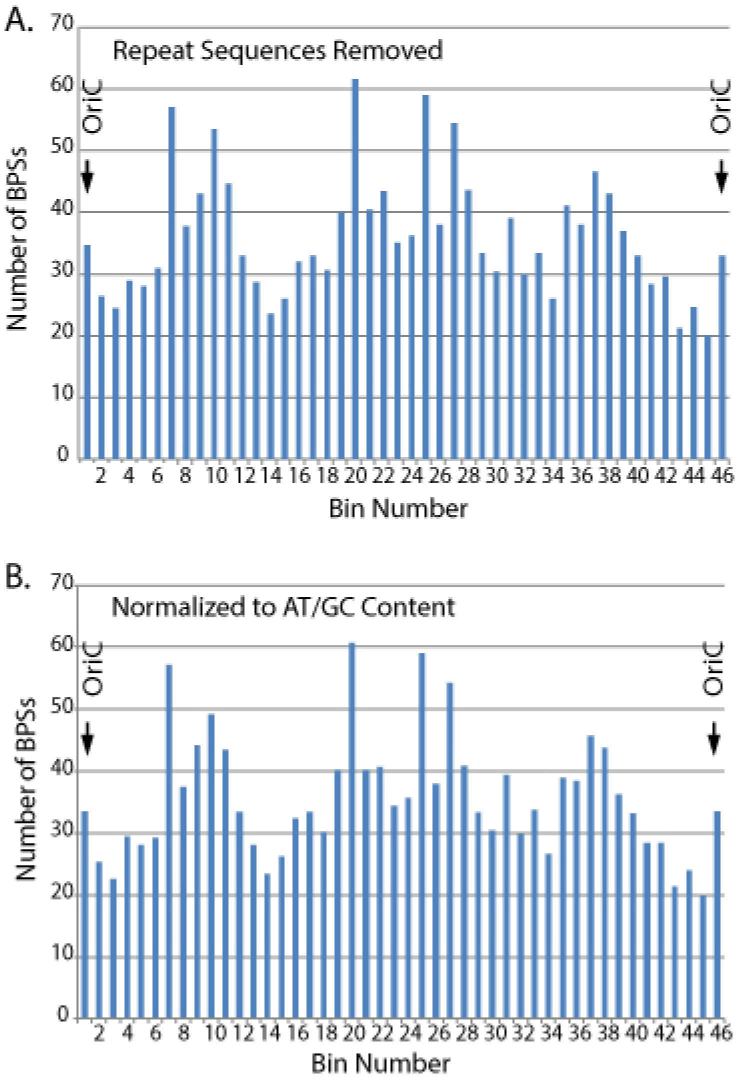

Figure S1 The stability of the pattern of BPS density to normalization. (A) Removal of repeated elements does not change the pattern of mutational density. The numbers of nucleotides in large repeated elements (IS elements and rRNA operons) were subtracted from the total nucleotides in each bin in which they occurred, and the mutations in each bin normalized to this new number. The histogram shows this adjusted number of mutations per bin with the bins starting at OriC. There is no difference between this pattern and the pattern without normalization shown in Figure 3A (χ^2 = 1.79, p = 1.00). (B) The pattern of mutational density across the genome is not due to the distribution of A:T and G:C base pairs. The numbers of mutations that occurred at A:T and at G:C base pairs have been normalized to the A:T and G:C content of each bin. The histogram shows this adjusted number of mutations per bin with the bins starting at OriC. The pattern of mutations per bin after this normalization matches the pattern without normalization shown in Figure 3A (χ^2 = 0.27, p = 1.00).

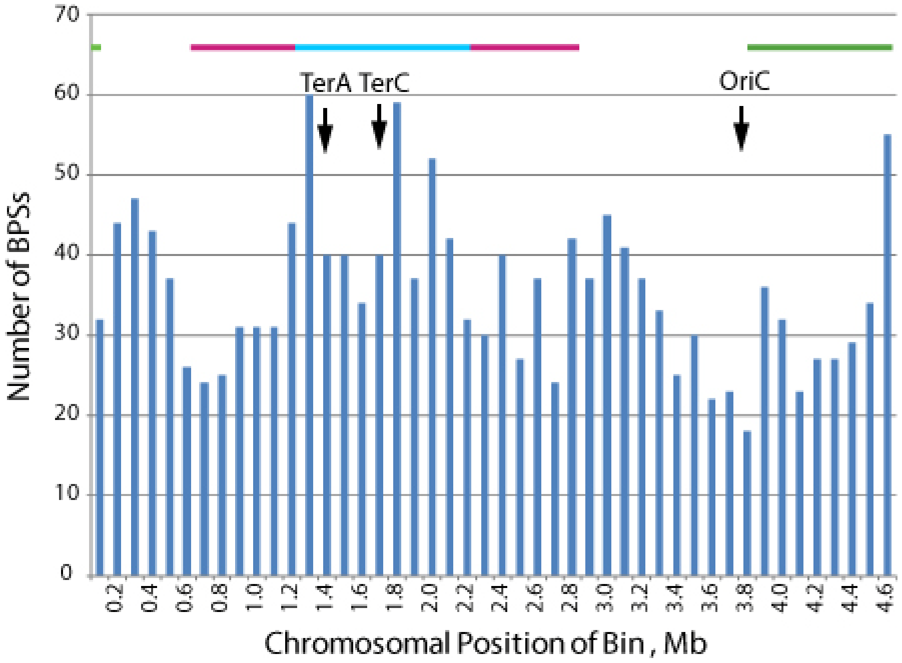

Figure S2 The stability of the pattern of BPS density after bin displacement. The 1625 BPSs mutations that accumulated in the MutL^{-} strain were collected into 46 bins, as in Figure 3A, but the bins start at the traditional zero point of the *E. coli* chromosome (which is in bin 8 in Figure 3A). The numbers on the X-axis give the end point of each bin in Mb. OriC = the origin of replication at 3924 Kb; TerA and TerC = strong termination sites at 1340 Kb and 1607 Kb (Duggin & Bell 2009). The four macrodomains (MDs) defined by the efficiency of recombinational exchange within each domain (Niki *et al.* 2000; Valens *et al.* 2004) are indicated: green = Ori MD, cherry = Left MD and Right MD, cyan = Terminal MD.

Table S1 The nucleotide coordinates of forty-six equally-sized bins starting at the origin of replication

Right Replichore[a]				Left Replichore[a]			
Bin #	Bin Coordinates, Nt		No. of BPS	Bin #	Bin Coordinates, Nt		No. of BPS
	Start	End			Start	End	
1	3,923,882	4,024,744	33	24	1,604,045	1,704,907	36
2	4,024,745	4,125,607	25	25	1,704,908	1,805,770	59
3	4,125,608	4,226,470	22	26	1,805,771	1,906,633	38
4	4,226,471	4,327,333	29	27	1,906,634	2,007,496	54
5	4,327,334	4,428,196	28	28	2,007,497	2,108,359	42
6	4,428,197	4,529,059	29	29	2,108,360	2,209,222	33
7	4,529,060	4,629,922	57	30	2,209,223	2,310,085	30
8[a]	4,629,923	91113	37	31	2,310,086	2,410,948	39
9	91,114	191,976	43	32	2,410,949	2,511,811	30
10	191,977	292,839	48	33	2,511,812	2,612,674	33
11	292,840	393,702	43	34	2,612,675	2,713,537	26
12	393,703	494,565	34	35	2,713,538	2,814,400	39
13	494,566	595,428	27	36	2,814,401	2,915,263	37
14	595,429	696,291	23	37	2,915,264	3,016,126	47
15	696,292	797,154	26	38	3,016,127	3,116,989	43
16	797,155	898,017	32	39	3,116,990	3,217,852	36
17	898,018	998,880	33	40	3,217,853	3,318,715	33
18	998,881	1,099,743	30	41	3,318,716	3,419,578	28
19	1,099,744	1,200,606	40	42	3,419,579	3,520,441	28
20	1,200,607	1,301,469	61	43	3,520,442	3,621,304	21
21	1,301,470	1,402,332	40	44	3,621,305	3,722,167	23
22	1,402,333	1,503,195	41	45	3,722,168	3,823,030	21
23	1,503,196	1,604,058	35	46	3,823,031	3,923,893	33

[a] The reference genome sequence was NC_000913.2 (MG1655). The origin of replication extends for about 230 nt; we have taken the midpoint, nt 3,923,882, as the start of the bins. The chromosome is 4,639,675 nt long; bin 8 includes the traditional zero point. There are a total of 1625 BPSs.

Table S2 Non-significant correlations of the numbers of mutations per bin with various genomic features

Feature	Pearson's product-moment correlation coefficient[a]			Data reference
	ρ_p	*p*	*q*	
**Gene Expression**				
No. of genes	0.175	0.245	0.180	NCBI[b]
No. of expressed genes, microarray data[c]	0.171	0.255	0.180	(Allen *et al.* 2006)
" " minus ribosomal protein genes[d]	0.256	0.086	0.100	"
Average gene expression, microarray data[e]	-0.247	0.100	0.106	(Jeong *et al.* 2004)
" " minus ribosomal protein genes[d]	-0.254	0.089	0.100	"
Average gene expression, RNA-Seq data	0.088	0.563	0.329	(Martincorena *et al.* 2012)
" " minus outlier[f]	0.068	0.658	0.348	"
**Factor Binding Sites[g]**				
Global transcription factor binding sites	0.004	0.977	0.435	Regulon DB
FIS Binding Sites	0.046	0.763	0.380	"
IHF Binding sites	0.009	0.950	0.435	"
LRP Binding sites	0.219[h]	0.145	0.136	"
H-NS Binding sites	0.177	0.240	0.179	"
Chi sites (forward in each replichore)	-0.158	0.295	0.200	This report[b]
**Genomic Structural Features**				
No. of H-NS sensitive genes (mid-log)	-0.020	0.896	0.433	(Blot *et al.* 2006)
" " upregulated in H-NS$^-$ mutant	0.191	0.205	0.165	"
" " downregulated in H-NS$^-$ mutant	-0.285	0.055	0.078	"
H-NS response per gene (mid-log)	-0.208	0.166	0.148	"
FIS response per gene (mid-log)	-0.073	0.632	0.345	"
No. of supercoiling sensitive genes	0.225	0.132	0.131	(Peter *et al.* 2004)
" " relaxation repressed	0.191	0.204	0.165	"
" " relaxation induced	0.077	0.609	0.344	"
Supercoiling response per gene	0.014	0.926	0.435	"
Gyrase response per gene[i]	-0.153	0.310	0.202	(Jeong *et al.* 2004)

[a]ρ_p is the correlation coefficient; *p* is a measure of the false positive rate; *q* is a measure of the false discovery rate appropriate for evaluating multiple comparisons (see Material and Methods). [b]The reference genome sequence was NC_000913.2 (MG1655). [c]Data obtained from http://bigg.ucsd.edu (Schellenberger *et al.* 2010). The correlations were not improved by considering the direction of transcription of the genes relative to the replication fork movement. [d]Twenty-eight highly expressed ribosomal protein genes in bin 42 were eliminated for this calculation. [e]Data obtained from http://www.ncbi.nlm.nih.gov/geo/. The correlations were not improved by considering the direction of transcription of the genes relative to the replication fork movement. [f]Bin 35 was eliminated for this calculation because the value of gene expression in it was more than two standard deviations greater than the mean. [g]FIS, IHF, LRP, H-NS, and HU are global transcriptional factors and nucleoid-associated proteins; Chi sites are recombination facilitating sites. [h]This correlation is due to one point; eliminating bin 20 reduces the correlation to -0.024. [i]Data obtained from http://www.ncbi.nlm.nih.gov/geo/. Values are the average per bin of the ratio of gene expression in a wild-type strain to that in a *gyrA* mutant.

Table S3 Genomic features used in linear regressions with the mutational data[a]

Feature[b]	Correlation with mutational data ρ_p	Value in 2-factor model		Value in 5-factor model		Data Reference
		Value	SE	Value	SE	
**Sequence Features**						
A:T content	0.325	0		0		NCBI
Average gene CAI	-0.373	0		-96.7	53.7	(Puigbo *et al.* 2008)
No. of genes downregulated in HU⁻ mutant	0.387	0		0		(Berger *et al.* 2010)
HU response per gene minus *hupAB*	0.455	7.8	2.7	5.0	3.0	"
Gyrase binding distribution	-0.360	0		0		(Jeong *et al.* 2004)
No. of Gyrase sensitive genes	0.382	0		0		"
No. of genes downregulated in *gyrA* mutant	0.481	0		0		"
No. of FIS sensitive genes (mid-log)	0.365	0		0		(Blot *et al.* 2006)
No. of genes upregulated in Fis⁻ mutant	0.457	1.5	0.5	1.0	0.5	"
SeqA binding sites	-0.322	0		0		(Sanchez-Romero *et al.* 2010)
**Additional Features[c]**						
No. of genes	0.175	NA		0		*NCBI
No. of expressed genes minus ribosomal protein genes	0.256	NA		0		((Allen *et al.* 2006))
Average gene expression minus ribosomal protein genes	-0.254	NA		0		(Jeong *et al.* 2004)
No. of genes downregulated in H-NS⁻ mutant	-0.285	NA		-0.8	0.5	(Blot *et al.* 2006)
H-NS response per gene (mid-log)	-0.208	NA		0		"
No. of supercoiling sensitive genes	0.225	NA		0		(Peter *et al.* 2004)
No. of relaxation repressed genes	0.190	NA		0.9	0.4	"

[a]See Table 1 and Table S1 for more information; SE = standard error; NA = not applicable; ρ_p = Pearson's product-moment correlation coefficient. [b]Only the first ten features were used for a linear regression that generated the 2-factor model (Figure 4B). [c]These seven additional features were added to the first ten features for a linear regression that generated the 5-factor model (Figure 4C).
