# Supplementary material for: On the Mutational Topology of the Bacterial Genome
Source: G3 (Bethesda). 2013 Mar 1;3(3):399–407. doi: 10.1534/g3.112.005355 (PMC3583449; doi:10.1534/g3.112.005355)
Supplement: Supporting Information [file supp_3.3.399_FigureS1.pdf]

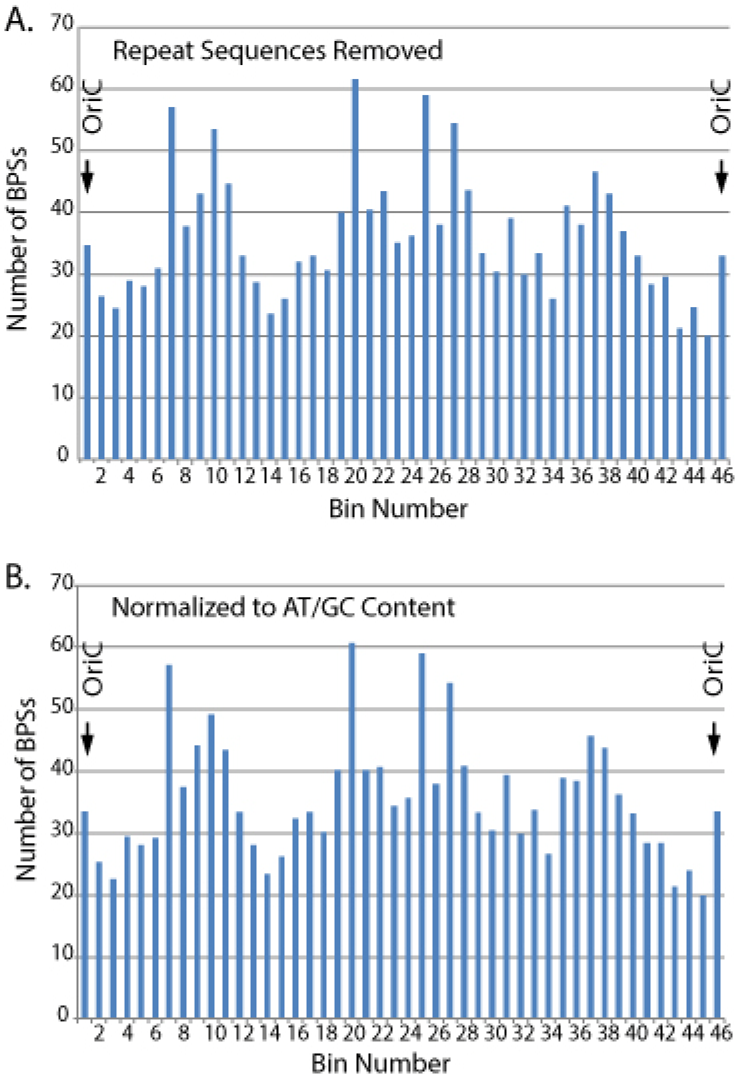

Figure S1 The stability of the pattern of BPS density to normalization. (A) Removal of repeated elements does not change the pattern of mutational density. The numbers of nucleotides in large repeated elements (IS elements and rRNA operons) were subtracted from the total nucleotides in each bin in which they occurred, and the mutations in each bin normalized to this new number. The histogram shows this adjusted number of mutations per bin with the bins starting at OriC. There is no difference between this pattern and the pattern without normalization shown in Figure 3A ($\chi^2 = 1.79$, $p = 1.00$). (B) The pattern of mutational density across the genome is not due to the distribution of A:T and G:C base pairs. The numbers of mutations that occurred at A:T and at G:C base pairs have been normalized to the A:T and G:C content of each bin. The histogram shows this adjusted number of mutations per bin with the bins starting at OriC. The pattern of mutations per bin after this normalization matches the pattern without normalization shown in Figure 3A ($\chi^2 = 0.27$, $p = 1.00$).
