# Supplementary material for: On the Mutational Topology of the Bacterial Genome
Source: G3 (Bethesda). 2013 Mar 1;3(3):399–407. doi: 10.1534/g3.112.005355 (PMC3583449; doi:10.1534/g3.112.005355)
Supplement: Supporting Information [file supp_3.3.399_FigureS2.pdf]

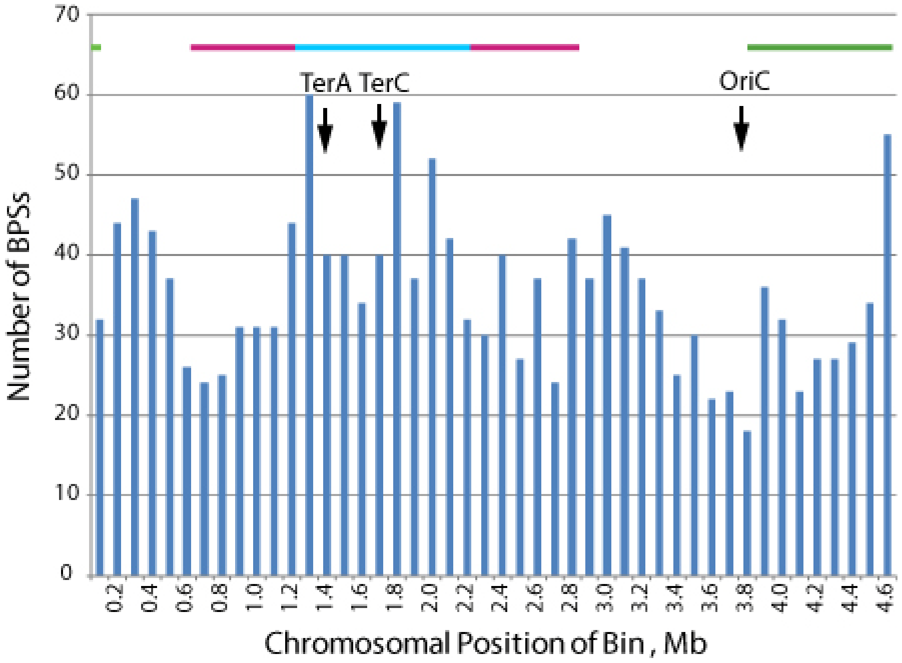

Figure S2 The stability of the pattern of BPS density after bin displacement. The 1625 BPSs mutations that accumulated in the MutL^{-} strain were collected into 46 bins, as in Figure 3A, but the bins start at the traditional zero point of the *E. coli* chromosome (which is in bin 8 in Figure 3A). The numbers on the X-axis give the end point of each bin in Mb. OriC = the origin of replication at 3924 Kb; TerA and TerC = strong termination sites at 1340 Kb and 1607 Kb (Duggin & Bell 2009). The four macrodomains (MDs) defined by the efficiency of recombinational exchange within each domain (Niki *et al.* 2000; Valens *et al.* 2004) are indicated: green = Ori MD, cherry = Left MD and Right MD, cyan = Terminal MD.
