# Supplementary material for: On the Mutational Topology of the Bacterial Genome
Source: G3 (Bethesda). 2013 Mar 1;3(3):399–407. doi: 10.1534/g3.112.005355 (PMC3583449; doi:10.1534/g3.112.005355)
Supplement: Supporting Information [file supp_3.3.399_TableS1.pdf]

Table S1 The nucleotide coordinates of forty-six equally-sized bins starting at the origin of replication

Right Replichore[a]				Left Replichore[a]			
Bin #	Bin Coordinates, Nt		No. of BPS	Bin #	Bin Coordinates, Nt		No. of BPS
	Start	End			Start	End	
1	3,923,882	4,024,744	33	24	1,604,045	1,704,907	36
2	4,024,745	4,125,607	25	25	1,704,908	1,805,770	59
3	4,125,608	4,226,470	22	26	1,805,771	1,906,633	38
4	4,226,471	4,327,333	29	27	1,906,634	2,007,496	54
5	4,327,334	4,428,196	28	28	2,007,497	2,108,359	42
6	4,428,197	4,529,059	29	29	2,108,360	2,209,222	33
7	4,529,060	4,629,922	57	30	2,209,223	2,310,085	30
8[a]	4,629,923	91113	37	31	2,310,086	2,410,948	39
9	91,114	191,976	43	32	2,410,949	2,511,811	30
10	191,977	292,839	48	33	2,511,812	2,612,674	33
11	292,840	393,702	43	34	2,612,675	2,713,537	26
12	393,703	494,565	34	35	2,713,538	2,814,400	39
13	494,566	595,428	27	36	2,814,401	2,915,263	37
14	595,429	696,291	23	37	2,915,264	3,016,126	47
15	696,292	797,154	26	38	3,016,127	3,116,989	43
16	797,155	898,017	32	39	3,116,990	3,217,852	36
17	898,018	998,880	33	40	3,217,853	3,318,715	33
18	998,881	1,099,743	30	41	3,318,716	3,419,578	28
19	1,099,744	1,200,606	40	42	3,419,579	3,520,441	28
20	1,200,607	1,301,469	61	43	3,520,442	3,621,304	21
21	1,301,470	1,402,332	40	44	3,621,305	3,722,167	23
22	1,402,333	1,503,195	41	45	3,722,168	3,823,030	21
23	1,503,196	1,604,058	35	46	3,823,031	3,923,893	33

[a] The reference genome sequence was NC_000913.2 (MG1655). The origin of replication extends for about 230 nt; we have taken the midpoint, nt 3,923,882, as the start of the bins. The chromosome is 4,639,675 nt long; bin 8 includes the traditional zero point. There are a total of 1625 BPSs.
