# Supplementary material for: On the Mutational Topology of the Bacterial Genome
Source: G3 (Bethesda). 2013 Mar 1;3(3):399–407. doi: 10.1534/g3.112.005355 (PMC3583449; doi:10.1534/g3.112.005355)
Supplement: Supporting Information [file supp_3.3.399_TableS2.pdf]

Table S2 Non-significant correlations of the numbers of mutations per bin with various genomic features

Feature	Pearson's product-moment correlation coefficient[a]			Data reference
	ρ_p	*p*	*q*	
**Gene Expression**				
No. of genes	0.175	0.245	0.180	NCBI[b]
No. of expressed genes, microarray data[c]	0.171	0.255	0.180	(Allen *et al.* 2006)
" " minus ribosomal protein genes[d]	0.256	0.086	0.100	"
Average gene expression, microarray data[e]	-0.247	0.100	0.106	(Jeong *et al.* 2004)
" " minus ribosomal protein genes[d]	-0.254	0.089	0.100	"
Average gene expression, RNA-Seq data	0.088	0.563	0.329	(Martincorena *et al.* 2012)
" " minus outlier[f]	0.068	0.658	0.348	"
**Factor Binding Sites[g]**				
Global transcription factor binding sites	0.004	0.977	0.435	Regulon DB
FIS Binding Sites	0.046	0.763	0.380	"
IHF Binding sites	0.009	0.950	0.435	"
LRP Binding sites	0.219[h]	0.145	0.136	"
H-NS Binding sites	0.177	0.240	0.179	"
Chi sites (forward in each replichore)	-0.158	0.295	0.200	This report[b]
**Genomic Structural Features**				
No. of H-NS sensitive genes (mid-log)	-0.020	0.896	0.433	(Blot *et al.* 2006)
" " upregulated in H-NS$^-$ mutant	0.191	0.205	0.165	"
" " downregulated in H-NS$^-$ mutant	-0.285	0.055	0.078	"
H-NS response per gene (mid-log)	-0.208	0.166	0.148	"
FIS response per gene (mid-log)	-0.073	0.632	0.345	"
No. of supercoiling sensitive genes	0.225	0.132	0.131	(Peter *et al.* 2004)
" " relaxation repressed	0.191	0.204	0.165	"
" " relaxation induced	0.077	0.609	0.344	"
Supercoiling response per gene	0.014	0.926	0.435	"
Gyrase response per gene[i]	-0.153	0.310	0.202	(Jeong *et al.* 2004)

[a]ρ_p is the correlation coefficient; *p* is a measure of the false positive rate; *q* is a measure of the false discovery rate appropriate for evaluating multiple comparisons (see Material and Methods). [b]The reference genome sequence was NC_000913.2 (MG1655). [c]Data obtained from http://bigg.ucsd.edu (Schellenberger *et al.* 2010). The correlations were not improved by considering the direction of transcription of the genes relative to the replication fork movement. [d]Twenty-eight highly expressed ribosomal protein genes in bin 42 were eliminated for this calculation. [e]Data obtained from http://www.ncbi.nlm.nih.gov/geo/. The correlations were not improved by considering the direction of transcription of the genes relative to the replication fork movement. [f]Bin 35 was eliminated for this calculation because the value of gene expression in it was more than two standard deviations greater than the mean. [g]FIS, IHF, LRP, H-NS, and HU are global transcriptional factors and nucleoid-associated proteins; Chi sites are recombination facilitating sites. [h]This correlation is due to one point; eliminating bin 20 reduces the correlation to -0.024. [i]Data obtained from http://www.ncbi.nlm.nih.gov/geo/. Values are the average per bin of the ratio of gene expression in a wild-type strain to that in a *gyrA* mutant.
