# Supplementary material for: On the Mutational Topology of the Bacterial Genome
Source: G3 (Bethesda). 2013 Mar 1;3(3):399–407. doi: 10.1534/g3.112.005355 (PMC3583449; doi:10.1534/g3.112.005355)
Supplement: Supporting Information [file supp_3.3.399_TableS3.pdf]

Table S3 Genomic features used in linear regressions with the mutational data[a]

Feature[b]	Correlation with mutational data ρ_p	Value in 2-factor model		Value in 5-factor model		Data Reference
		Value	SE	Value	SE	
**Sequence Features**						
A:T content	0.325	0		0		NCBI
Average gene CAI	-0.373	0		-96.7	53.7	(Puigbo *et al.* 2008)
No. of genes downregulated in HU$^-$ mutant	0.387	0		0		(Berger *et al.* 2010)
HU response per gene minus *hupAB*	0.455	7.8	2.7	5.0	3.0	"
Gyrase binding distribution	-0.360	0		0		(Jeong *et al.* 2004)
No. of Gyrase sensitive genes	0.382	0		0		"
No. of genes downregulated in *gyrA* mutant	0.481	0		0		"
No. of FIS sensitive genes (mid-log)	0.365	0		0		(Blot *et al.* 2006)
No. of genes upregulated in Fis$^-$ mutant	0.457	1.5	0.5	1.0	0.5	"
SeqA binding sites	-0.322	0		0		(Sanchez-Romero *et al.* 2010)
**Additional Features[c]**						
No. of genes	0.175	NA		0		*NCBI
No. of expressed genes minus ribosomal protein genes	0.256	NA		0		((Allen *et al.* 2006))
Average gene expression minus ribosomal protein genes	-0.254	NA		0		(Jeong *et al.* 2004)
No. of genes downregulated in H-NS$^-$ mutant	-0.285	NA		-0.8	0.5	(Blot *et al.* 2006)
H-NS response per gene (mid-log)	-0.208	NA		0		"
No. of supercoiling sensitive genes	0.225	NA		0		(Peter *et al.* 2004)
No. of relaxation repressed genes	0.190	NA		0.9	0.4	"

[a]See Table 1 and Table S1 for more information; SE = standard error; NA = not applicable; ρ_p = Pearson's product-moment correlation coefficient. [b]Only the first ten features were used for a linear regression that generated the 2-factor model (Figure 4B). [c]These seven additional features were added to the first ten features for a linear regression that generated the 5-factor model (Figure 4C).
